# Prognostic relevance of *KRAS* genotype in metastatic colorectal cancer patients unfit for FIr-B/FOx intensive regimen

**DOI:** 10.3892/ijo.2014.2369

**Published:** 2014-04-04

**Authors:** GEMMA BRUERA, KATIA CANNITA, ALDO VICTOR GIORDANO, ROBERTO VICENTINI, CORRADO FICORELLA, ENRICO RICEVUTO

**Affiliations:** 1 Medical Oncology, University of L’Aquila, I-67100 L’Aquila, Italy; 2 Radiology, University of L’Aquila, I-67100 L’Aquila, Italy; 3 Hepatobiliar-Pancreatic Surgery, S. Salvatore Hospital, University of L’Aquila, I-67100 L’Aquila, Italy; 4 Department of Biotechnological and Applied Clinical Sciences, University of L’Aquila, I-67100 L’Aquila, Italy

**Keywords:** c.35 G>A *KRAS* mutation, elderly, FIr-B/FOx, *KRAS* genotype, metastatic colorectal cancer, triplet chemotherapy plus bevacizumab

## Abstract

First-line triplet chemotherapy plus bevacizumab (FIr-B/FOx) can improve efficacy of metastatic colorectal cancer (MCRC), *KRAS* wild-type and mutant. Prognostic relevance of *KRAS* genotype was evaluated in patients unfit for FIr-B/FOx, treated with conventional medical treatments. Consecutive MCRC patients not eligible for FIr-B/FOx regimen due to age (≥75 years) and/or comorbidities were treated with tailored conventional first-line treatments. *KRAS* codon 12/13 mutations were screened by direct sequencing. Activity and efficacy were evaluated and compared according to medical treatments, age (non-elderly and elderly ≥65 years), comorbidity stage (Cumulative Illness Rating Scale), metastatic extension (liver-limited and other/multiple metastatic), and *KRAS* genotype, using log-rank. Selected first line treatments were medical in 37 patients (92.5%), and surgical in 3 patients (7.5%). Medical treatment regimens: triplet, 18 (45%); doublet, 15 (37.5%); mono-therapy, 4 (10%). At median follow-up of 8 months, objective response rate (ORR) was 37%, median progression-free survival (PFS) 7 months, liver metastasectomies 8% (liver-limited disease 37.5%), median overall survival (OS) 13 months. Triplet regimens failed to significantly affect clinical outcome, compared to doublet. According to *KRAS* genotype, ORR, PFS and OS were, respectively: wild-type 50%, 8 months, 13 months; mutant 25%, 6 months, 9 months. *KRAS* genotype wild-type compared to mutant significantly affected PFS, while not OS. *KRAS* c.35 G>A mutation (G12D) significantly affected worse PFS and OS compared to wild-type and/or other mutations. *KRAS* genotype, specifically the c.35 G>A *KRAS* mutation, may indicate poor prognosis in MCRC patients unfit for intensive medical treatments.

## Introduction

Clinical management of metastatic colorectal cancer (MCRC) faces with different options and lines of treatment according to patients’ fitness [age, performance status (PS), comorbidities], extension of metastatic disease [liver-limited (L-L) or other/multiple metastatic (O/MM)], *KRAS* genotype (1–4). First line triplet chemotherapy, or doublets plus bevacizumab (BEV) or cetuximab, reported overlapping activity and efficacy in phase III trials, ranging between ORR 39–68%, PFS 7.2–10.6 months and OS 19.9–26.1 months (2,5,6). More intensive regimens, consisting of triplet chemotherapy plus targeted agents, can further increase activity, efficacy and effectiveness of liver metastasectomies (7–9).

In clinical practice, a decision-making process including functional, nutritional, and co-morbidity status is required to tailor first line medical treatment (10). Elderly status (age >65 years), PS >2, and/or comorbidities represent major features, to limit toxicities and maintain quality of life (QoL). Elderly MCRC patients are prevalent, and a clinical challenge is to select between intensive or tailored medical treatments, by properly weighing expected safety and efficacy, and according to prognostic factors. Retrospective studies showed that elderly patients benefit from 5-fluorouracil (5-FU) (11–13), irinotecan (CPT-11)-containing therapy (14,15), FOLFOX (16) to the same extent as younger (17–19). In the OPTIMOX1 trial, ORR 59%, PFS 9.0 months and OS 20.7 months were comparable between old-elderly and younger patients treated with FOLFOX (20). Treatment efficacy was also comparable with BEV associated to 5-FU/CPT-11 (21). In elderly patients, addition of BEV to 5-FU based chemotherapy significantly prolonged PFS (9.2-9.3 months) and OS (17.4–19.3 months) (22,23). In BRiTE and BEAT studies, no different PFS was observed in elderly patients; median OS decreased with age (24,25). In the randomized phase III trial comparing FOLFIRI with FOLFOXIRI, age was not significantly related to activity and efficacy, with OS 16.9 and 19.9 months, respectively (26,27). ORR was significantly lower in older patients treated with FOLFOXIRI (27). Patients underwent metastasectomies without increased morbidity or mortality, irrespective of age. Patients with PS 2 presented a significantly lower OS and PFS, irrespectively of FOLFIRI or FOLFOXIRI chemotherapy regimen (27). Age and/or comorbidities did not affect efficacy in patients treated with cetuximab added to FOLFOX or FOLFIRI (28). In elderly and PS 2 patients, PFS was not increased by addition of panitumumab to FOLFOX (29). A meta-analysis showed that PS 1 compared to PS 2 significantly affect prognosis, regardless of treatment, with ORR 43.8 vs 32%, PFS 7.6 vs 4.9 months, OS 17.3 and 8.5 months, respectively (30). The FOCUS2 randomized trial prospectively evaluated first line chemotherapy options consisting of 80% dose 5-FU or capecitabine, with or without oxaliplatin (OXP), in old-elderly and/or frail patients, and showed that addition of OXP significantly improved ORR (35 vs 13%), a trend of PFS (5.8 vs. 4.5 months, hazard ratio 0.84, p=0.07), but not OS (31), without significantly increasing toxicity, with a negative impact on QoL.

*KRAS* mutations occur in 35–45% of colorectal cancer (CRC), mostly codon 12 (80%), prevalently c.35 G>A (G12D) transversion (32.5%) (32,33), impairing the intrinsic GTPase activity, and leading to constitutive, growth factor receptor-independent activation of downstream signalling (34). In the *in vitro* model proposed by Guerrero *et al* (35), codon 12 mutations increase aggressiveness by the differential regulation of KRAS downstream pathways that lead to inhibition of apoptosis, enhanced loss of contact inhibition and increased predisposition to anchorage-independent growth. *KRAS* genotype, wild-type or mutant, addresses the addition of targeted agents in MCRC medical treatment: anti-EGFR or anti-VEGF to doublet chemotherapy in *KRAS* wild-type (36–39); BEV to 5-FU, CPT-11 in *KRAS* mutant, significantly predicting prolonged PFS, while not OS and activity (36,37).

Clinical outcome in wild-type and mutant patients assesses the prognostic relevance of *KRAS* genotype, depending on differential tumor biological aggressiveness (4), including the predictive effectiveness of treatment strategies. Median OS of patients treated with BEV added to CPT-11/5-FU or triplet chemotherapy was different in *KRAS* wild-type and mutant patients, but not significantly (4,8,36,37); *KRAS* wild-type L-L patients may achieve a significantly greater benefit from integration with liver metastasectomies, with respect to mutant patients (4). We recently reported that the prevalent *KRAS* c.35 G>A (G12D) mutant genotype may significantly affect worse OS of MCRC patients treated with FIr-B/FOx, compared to wild-type or different other mutations (40). Here, we report a retrospective exploratory analysis evaluating tailored first line treatments, the prognostic value of *KRAS* genotype, and of the c.35 G>A mutation, in consecutive MCRC patients not eligible for intensive first line FIr-B/FOx expanded clinical program, due to age and/or comorbidities.

## Materials and methods

### 

#### Patient eligibility

Consecutive MCRC patients not eligible, due to comorbidities and/or age, for expanded clinical program or ongoing phase II trial proposing intensive regimens consisting of triplet chemotherapy plus targeted agent, were treated in clinical practice with first line medical and/or surgical treatments, chosen among those in indication for MCRC treatment and approved by Agenzia Italiana del Farmaco (AIFA) for administration *in label* in Italian public hospitals, and published in Gazzetta Ufficiale Repubblica Italiana (‘Elenco dei Medicinali erogabili a totale carico del Servizio sanitaria nazionale’, Gazzetta Ufficiale Repubblica Italiana N.1, 2 Gennaio 2009). Thus, it was not a clinical trial and approval by ethics committee and institutional review board was not necessary, because patients were treated with conventional treatments without any additional medical intervention out of the best common clinical practice. Patients had histological confirmed diagnosis of MCRC, age ≥18 years, PS ≤2. Criteria to define patients unfit, or not eligible for intensive regimens were: age ≥75 years; uncontrolled severe diseases; cardiovascular disease (uncontrolled hypertension, uncontrolled arrhythmia, ischemic cardiac diseases in the last year); thromboembolic disease, coagulopathy, preexisting bleeding diatheses; proteinuria >1 g/24 h. Patients were classified according to Cumulative Illness Rating Scale (CIRS) (10). Treatment options were tailored according to age (< or ≥75 years), patient’s fitness (PS, CIRS), *KRAS* genotype. Patients with PS 3 were not treated. All patients provided written, informed consent to the proposed *in label* treatment option.

### Methods

#### Medical treatment regimens

Medical treatments included triplet, doublet, or mono-chemotherapy. Triplet FIr/FOx schedule consisted of weekly timed-flat-infusion 5-FU (TFI 5-FU), associated to weekly alternating CPT-11 or L-OXP (41): TFI/5-FU (Fluorouracil Teva; Teva Italia, Milan, Italy), 750–900 mg/m^2^/die, over 12 h (from 10:00 pm to 10:00 am), days 1–2, 8–9, 15–16, 22–23; CPT-11 (Campto; Pfizer, Latina, Italy), 120–160 mg/m^2^, days 1 and 15; l-OXP (Eloxatin; Sanofi-Aventis, Milan, Italy), 70–80 mg/m^2^, days 8 and 22; cycles every 4 weeks. Other triplet, doublet and mono-regimens were administered according to previously reported schedules (7,41,42). Targeted agents were: BEV (Avastin; Roche, Welwyn Garden City, UK), 5 mg/kg, days 1 and 15; cetuximab (Erbitux; Merck, Darmstadt, Germany), 400 mg/m^2^ initial dose, then 250 mg/m^2^/week.

#### Mutational analysis

Genetic analyses were performed on paraffin-embedded tissue blocks from primary tumor and/or metastatic sites, as previously reported (4). Genotype status was assessed for *KRAS* codon 12 and 13 mutations by direct sequencing. *KRAS* exon 2 sequence was performed from PCR-amplified tumor DNA using the Big Dye V3.1 Terminator kit, electrophoresis in ABI PRISM 3130xl Genetic Analyzer, and analysis using the GeneMapper Analysis software version 4.0 (Applied Biosystems, Foster City, CA, USA).

#### Study design

Activity, efficacy, and prognostic relevance of first line treatments, and *KRAS* genotype on clinical outcomes were evaluated. Patients were classified according to: metastatic extension, L-L and O/MM (3,4); age, non-elderly (<65 years), young-elderly (≥65 <75 years), old-elderly (≥75 years); CIRS stage primary, intermediate, secondary. Clinical evaluation of response was made by CT scan; PET was added based on investigators’ assessment. Follow-up was scheduled every two-three months up to disease progression or death. L-L patients were evaluated at baseline and every two-three cycles of treatment by a multidisciplinary team, to evaluate resectability defined according to reported categories (3). Liver metastasectomies were defined as R0, if radical surgery, R1, if radioablation was added. Surgery was recommended >4 weeks after BEV discontinuation.

Clinical criteria of activity and efficacy were ORR, resection rate of metastases, PFS and OS: ORR, evaluated according to RECIST criteria (43); pathologic complete response, defined as no residual cancer cells in surgical specimens; PFS and OS, evaluated using the Kaplan-Meier method (44). PFS was defined as the length of time from the beginning of treatment and disease progression or death (resulting from any cause) or to the last contact; OS as the length of time between the beginning of treatment and death or to last contact. Log-rank test was used to compare PFS and OS according to medical treatment, *KRAS* genotype, metastatic extension, age and comorbidity stage (45).

## Results

### 

#### Patient demographics

Forty patients unfit for intensive regimens, among 72 consecutive MCRC (56%), were treated with (Table I): medical treatments, 37 patients (92.5%); surgery, 3 (7.5%). First line medical treatments: triplet, 18 (45%); doublet, 15 (37.5%); mono-therapy, 4 (10%). Among 39 *KRAS* evaluated patients (97.5%), 23 (59%) were wild-type and 16 (41%) mutant. Clinical features of the 37 patients who underwent first line medical treatments were (Table IIA): male/female ratio, 22/15; median age, 75 years; young- and old-elderly, 28 (76%) and 20 (54%), respectively; PS 0, 15 (41%) and 1–2, 22 (59%); metastatic disease metachronous 24%, synchronous 76%. Liver metastases, 26 patients (70%); L-L 8 (22%), O/MM 29 79%). Distribution of patients according to age and comorbidity stage (Table IIB): non-elderly 9 (24%), young-elderly 8 (22%), old-elderly 20 (54%); CIRS stage primary 4 (11%), intermediate 15 (40%), secondary 18 (42%). *KRAS* mutations detected in 15 patients were: codon 12, 13 (36.1%), specifically c.35 G>A (G12D), 7 (19.4%), c.35 G>T (G12V), 6 (16.6%); codon 13, 2 (5.5%), c.37 G>T (G13V), 1 (2.7%) and c.38 G>A (G13D), 1 (2.7%).

Medical treatments were tailored according to age and CIRS stage. Triplet regimens were administered in 18 patients (49%): non-elderly 6, young-elderly 4, old-elderly 8; CIRS primary 2, intermediate 10, secondary 6. Doublet regimens were administered in 15 patients (40%): non-elderly 3, young-elderly 3, old-elderly 9; CIRS primary 1, intermediate 4, secondary 10. Mono-regimens were administered in 4 patients (11%): young-elderly 1, old-elderly 3; CIRS primary 1, intermediate 1, secondary 2.

#### Overall activity and efficacy

**Figure 1.**
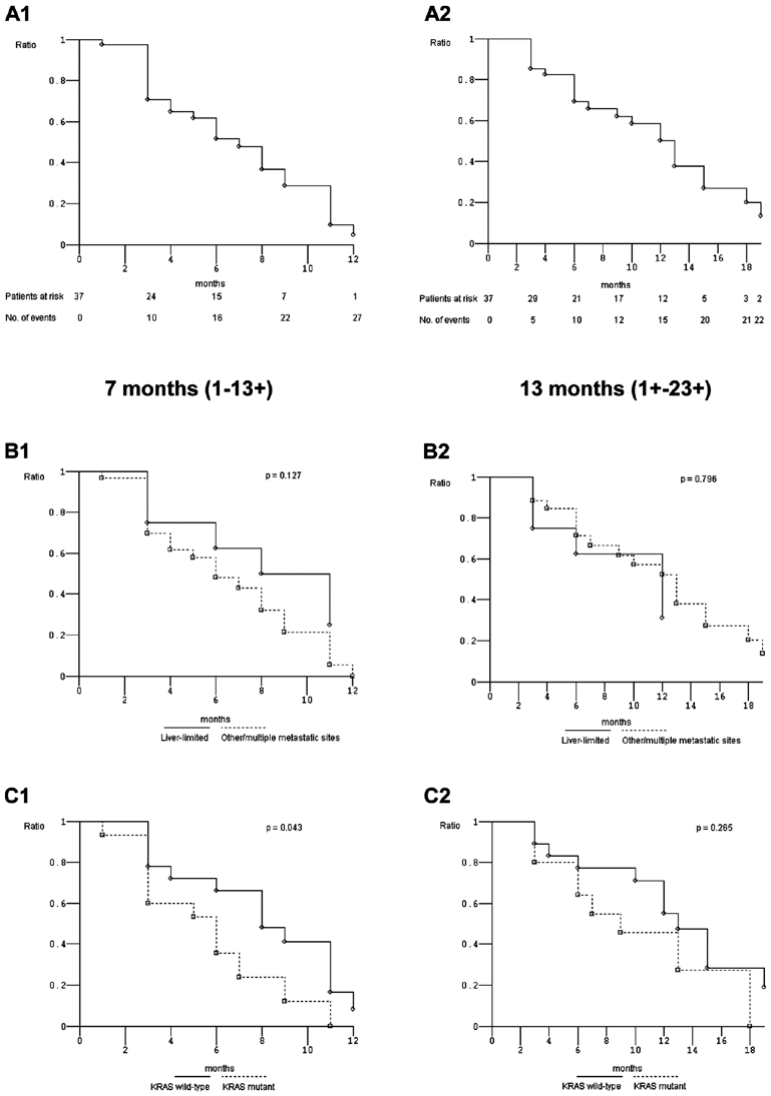
Kaplan-Meier survival estimate. (A) Overall treated patients; (B) L-L versus O/MM; (C) Overall population, *KRAS* wild-type versus *KRAS* mutant; (1) PFS; (2) OS.

Among the 37 patients who underwent medical treatments, 10 were not evaluable for activity: 7 (19%) did not receive at least 2 cycles of treatment; 3 were on-treatment. The intent-to-treat analysis of 27 patients showed ORR 37% (α 0.05, CI ± 19) (Table IIIA). We observed 10 objective responses: 9 partial (33%) and 1 complete (CR 4%); 9 stable diseases (33%); 8 progressive diseases (30%). Disease control rate was 67% (α 0.05, CI ± 18). After median follow-up of 8 months, median PFS was 7 months (1-13+): 28 events occurred. Median OS was 13 months (1+−23+): 22 events occurred (Fig. 1A). R0 liver metastasectomies were performed in 3 patients (8%): 3 out of 8 L-L (37.5%). No surgery-related complications were reported. Overall, 1 clinical plus 1 pathologic CR were reported (7%); 1 patient showed a progressive disease at 8 months; 1 patient was progression-free at 10 months. Pathologic CR was obtained in 1 *KRAS* wild-type patient (33%), with primary rectal tumor and a single L-L metastasis. Twelve patients (32%) received, at least, a second line treatment.

Among 7 evaluable L-L patients, ORR was 71%; 3 performed liver metastasectomies (43%) and 1 cCR (14%); median PFS 11 months (3–13+ months); median OS 12 months (3–13+ months). Among 20 evaluable O/MM patients, ORR was 25%; median PFS 6 months (1–12 months); median OS 13 months (1+−23+ months). Clinical outcome (PFS and OS) in L-L compared to O/MM patients was not significantly different (Fig. 1B).

#### Activity and efficacy according to first line treatment, elderly and comorbidity status

**Figure 2.**
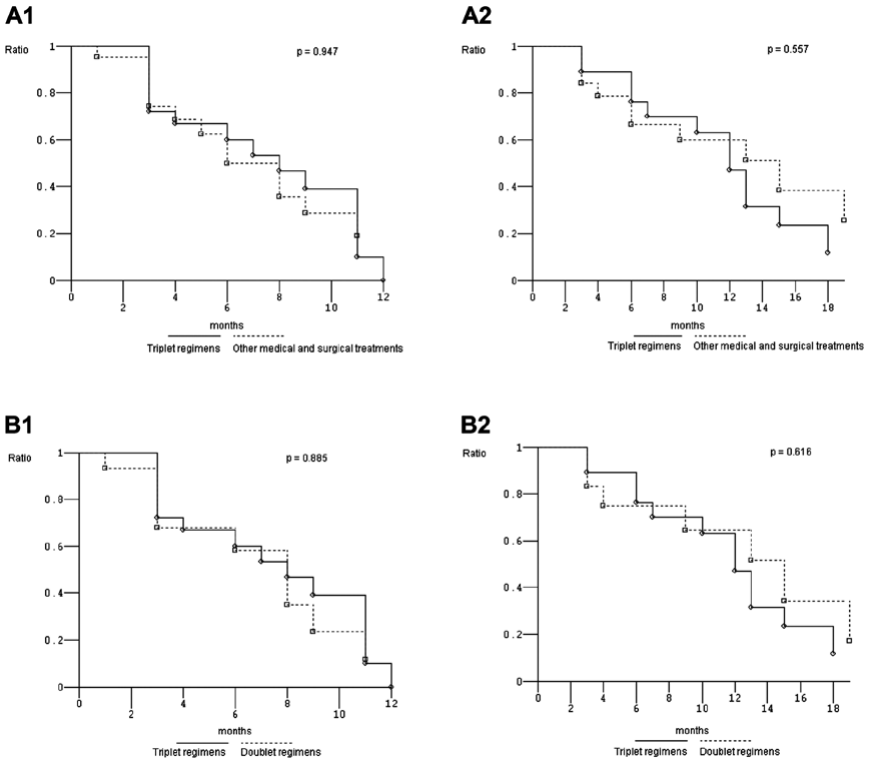
Kaplan-Meier survival estimate. (A) First line treatment, triplet regimens versus other medical and surgical treatments. (B) First line treatment, triplet regimens versus doublet regimens. (1) PFS; (2) OS.

Among 16 evaluable patients treated with triplet regimens (Table IIIB), ORR was 37.5% (α 0.05, CI ± 24). We observed 6 partial responses (37.5%); 5 stable diseases (31%); 5 progressive diseases (31%). Median PFS was 8 months (3–12): 14 events occurred. Median OS was 12 months (3–23+ months): 12 events occurred. Secondary metastasectomy was performed in 1 patient (6%). Among 15 patients treated with doublet regimens (Table IIIB), ORR was 44% (α 0.05, CI ± 34). We observed 3 partial responses (33%); 1 CR (11%); 3 stable diseases (33%); 2 progressive diseases (22%). Median PFS was 8 months (1–13+): 9 events occurred. Median OS was 15 months (1+−23+ months): 7 events occurred. Among 4 patients treated with mono-regimens, median PFS was 5 months (3–6 months), median OS 6 months (3−13+ months). Among 3 patients who underwent surgery as first line treatment, median PFS was not reached (3+−19+ months); median OS not reached (3+−19+ months). PFS and OS were not significantly different in patients treated with triplet compared to other first line treatments (p=0.947 and 0.557, respectively), and to doublet regimens (p= 0.885 and 0.616, respectively) (Fig. 2).

Moreover, PFS and OS were not significantly different in non-elderly and young-elderly compared to old-elderly patients (p=0.240 and 0.750, respectively), and in primary and intermediate CIRS stage compared to secondary stage patients (p=0.494 and 0.364, respectively).

#### Prognostic relevance of KRAS genotype and c.35 G>A KRAS mutation

**Figure 3.**
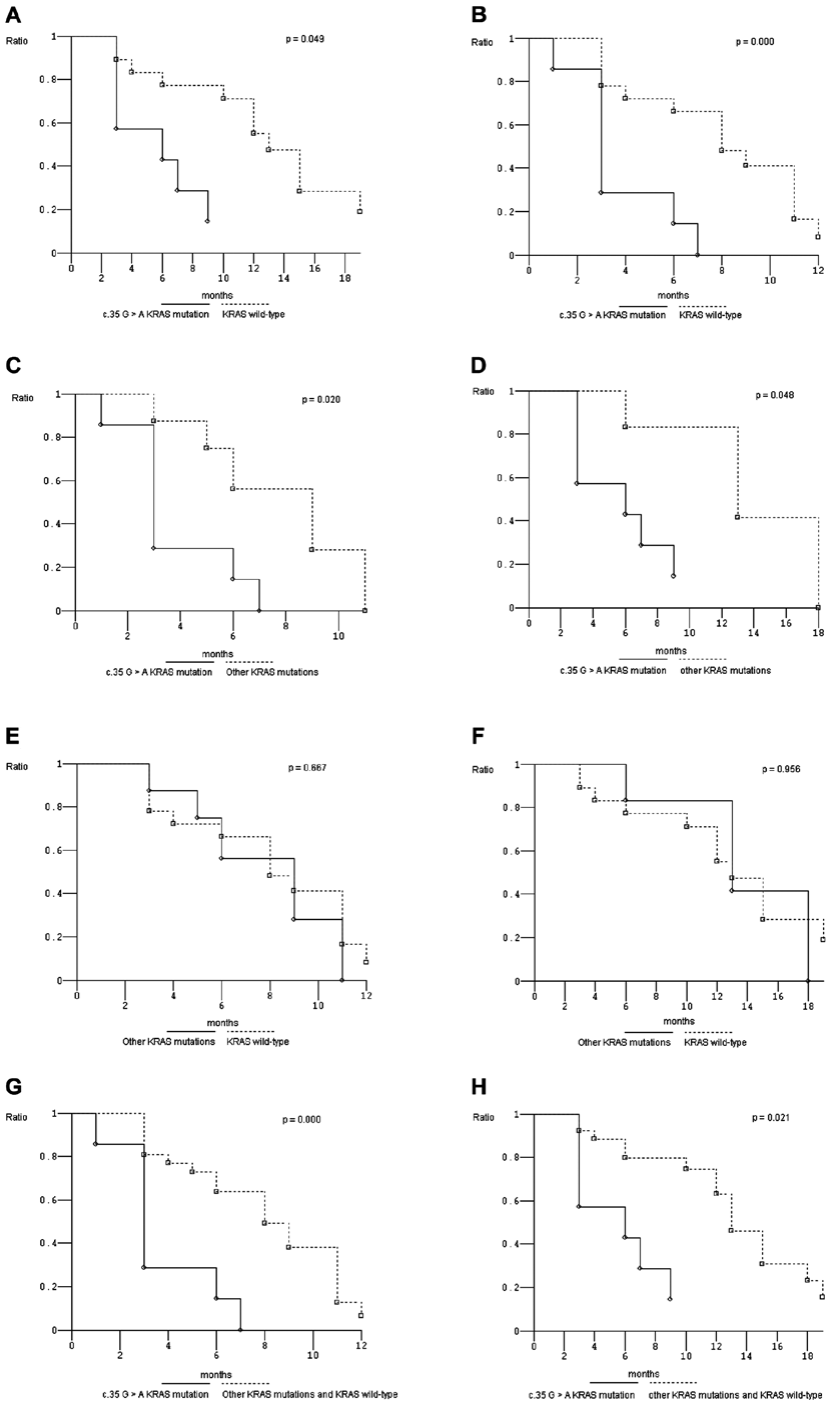
Kaplan-Meier survival estimate. (A) PFS c.35 G>A *KRAS* mutant versus wild-type patients; (B) OS c.35 G>A KRAS mutant versus wild-type patients; (C) PFS c.35 G>A *KRAS* mutant versus other mutant patients; (D) OS c.35 G>A *KRAS* mutant versus other mutant patients; (E) PFS other *KRAS* mutant versus wild-type patients; (F) OS other *KRAS* mutant versus wild-type patients; (G) PFS c.35 G>A *KRAS* mutant versus other mutant plus wild-type patients; (H) OS c.35 G>A *KRAS* mutant versus other mutant plus wild-type patients.

Among 14 *KRAS* wild-type patients evaluable for activity, ORR was 50% (α 0.05, CI ± 27) (Table IIIA). We observed 7 objective responses: 6 partial (43%) and 1 CR (7%); 4 stable diseases (29%); 3 progressive diseases (21%). Disease control rate was 79% (α 0.05, CI ± 22). Liver metastasectomies were performed in 3 patients (14%), 3 out of 7 L-L (43%). Median PFS was 8 months (1+−13+ months), 15 events occurred (71%). Median OS was 13 months (1+−23+ months), 11 events occurred. Among 12 *KRAS* mutant patients evaluable for activity, ORR was 25% (α 0.05, CI ± 26). We observed 3 partial responses (25%); 5 stable diseases (42%); 4 progressive diseases (33%). Disease control rate was 67% (α 0.05, CI ± 28). No liver metastasectomies were performed. Median PFS was 6 months (1–11 months), 12 events occurred (80%). Median OS was 8 months (3–18 months), 10 events occurred. *KRAS* wild-type compared with mutant patients showed significantly different PFS (p=0.043), but not OS (Fig. 1C). *KRAS* c.35 G>A mutant patients showed significantly worse PFS and OS compared to wild-type (p=0.000, and 0.049, respectively) (Fig. 3A and B), and to other mutant patients (p=0.020 and 0.048, respectively) (Fig. 3C and D). No different clinical outcomes were reported in other than c.35 G>A *KRAS* mutant compared to wild-type patients (Fig. 3E and F). PFS and OS were also significantly worse in c.35 G>A *KRAS* mutant patients compared to other mutant plus wild-type patients (p=0.000, and 0.021, respectively) (Fig. 3G and H).

## Discussion

Patients unfit for first line FIr-B/FOx intensive regimen, due to age (≥75 years) and/or comorbidities, were prevalent (56%), mostly elderly (76%), particularly old-elderly patients (54%), prevalently PS 1–2 (59%), CIRS stage intermediate/secondary (89%), O/MM disease (79%). Most unfit MCRC patients were treated with triplet or doublet regimens (49 and 40%, respectively), and some (19%) did not reach the first evaluation of activity at 2–3 months.

Retrospective evaluations of doublets consisting of CPT-11 or OXP, associated to 5-FU or capecitabine in elderly patients eligible for clinical trials gained ORR 18–59.4%, PFS 4.9–10.0 months and OS 8.5–20.7 months (11–16,20,30,31,46). BEV addition to 5-FU-based chemotherapy in elderly patients significantly increased PFS up to 9.2–9.3 months and OS up to 17.4–19.3 months (22,23). Triplet chemotherapy or doublets plus BEV obtained ORR 34.9–45.9%, PFS 7.9–9.3 months and OS 17.4–20.5 months (23–25). The present tailored approach, based on evaluation of elderly status and/or CIRS, prevalently addressing doublet and triplet regimens, reported ORR 37%, PFS 7 months and OS 13 months. Selected medical treatment, triplet compared to doublet, did not significantly affected PFS and OS, nor advanced age, or CIRS stage. The FOCUS2 randomized trial evaluating first line reduced dose 5-FU or capecitabine and OXP in old-elderly and/or frail patients showed significantly improved ORR 35%, with PFS 5.8 months (31). The meta-analysis evaluating the effect of PS on clinical outcome showed that PS 1 compared to PS 2 significantly affected prognosis, regardless of treatment, with ORR 43.8 vs 32%, PFS 7.6 vs 4.9 months, OS 17.3 and 8.5 months, respectively (30). In the HORG-FOLFOXIRI trial, elderly compared to non-elderly patients treated with FOLFIRI or FOLFOXIRI showed no different clinical outcome; significantly lower PFS and OS were reported in patients with PS 2 (26,27).

Young-elderly patients eligible for FIr-B/FOx intensive regimen, prevalently characterised by PS 0 (89%) and intermediate CIRS stage (93%), reported ORR 79%, PFS 11 months, OS 21 months, equivalent to overall patients (7,47). A complex decision-making process discriminating patients’ fitness, and tailoring a personalized medical treatment, is challenging: patients unfit for FIr-B/FOx can be treated with a two-drug first line combination regimen (31), but showed worse clinical outcome. No increased morbidity, nor mortality was reported in unfit patients who underwent secondary liver metastasectomies, reported as significantly higher in elderly patients (8%) (48).

Overall in MCRC patients treated with BEV added to CPT-11/5-FU, or with more intensive regimens (FIr-B/FOx, FOLFOXIRI/BEV), PFS and OS were not significantly different in *KRAS* wild-type and mutant (4,6,8,37) as well as in young-elderly patients (47). Recently, *KRAS* geno-type was reported as significantly affecting PFS and OS in patients treated with XelOx/BEV (49). We recently reported in MCRC patients treated with FIr-B/FOx, that the prevalent *KRAS* c.35 G>A (G12D) mutant genotype may significantly affect worse OS, compared to wild-type or other mutations (40). Present data reported for the first time that in patients unfit for FIr-B/FOx, *KRAS* wild-type compared to mutant patients showed a significantly different PFS, and not OS. Furthermore, *KRAS* c.35 G>A mutant genotype may affect significantly worse PFS and OS, compared to wild-type and/or other mutant, confirming that *KRAS* genotype, particularly c.35 G>A mutant, confers different biological aggressiveness (35), less effectively overcome by conventional triplet and doublet regimens. The prognostic relevance of *KRAS* genotype, particularly c.35 G>A mutant (4,40), and the predictive relevance of different medical treatments according to patients’ fitness for intensive regimens, should be prospectively evaluated.

In conclusion, in MCRC patients unfit for first line intensive FIr-B/FOx regimen, tailored doublet and triplet medical treatments showed similar activity and efficacy, also according to age and comorbidities. *KRAS* genotype may indicate different PFS, and c.35 G>A *KRAS* mutant a significantly worse PFS and OS, compared to wild-type and other mutations. Present findings warrant prospective trials comparing clinical outcome in unfit patients, according to *KRAS* genotype.

## Figures and Tables

**Table I.** First line clinical management of unfit MCRC patients.

	Overall	*KRAS* genotype	
	
	No. of patients (%)	Wild-type (%)	Mutant (%)
Total no.	40	23	16
Medical treatment	37 (92.5)	21 (91)	15 (94)
Triplet regimen	18 (45)	8 (35)	10 (62.5)
Doublet chemotherapy plus bevacizumab	3	1	2
Doublet chemotherapy plus cetuximab	5	5	-
Triplet chemotherapy	10	2	8
Doublet regimen	15 (37.5)	12 (52)	3 (19)
Mono-chemotherapy plus bevacizumab	2	-	2
Mono-chemotherapy plus cetuximab	8	8	-
Doublet chemotherapy	5	4	1
Mono-therapy	4 (10)	1 (4)	2 (12.5)
Mono-chemotherapy	4	1	2
Surgery	3	2	1

**Table:** A, Features of the unfit patients

	Overall treated Total no. (%)	*KRAS* wild-type Total no. (%)	*KRAS* mutant Total no. (%)
No. of patients	37	21 (58)	15 (42)
Gender			
Male/female	22/15	12/9	10/5
Age, years			
Median	75	77	69
Range	45–87	45–83	50–87
Elderly			
≥65 years	28 (76)	18 (86)	9 (60)
≥75 years	20 (54)	13 (62)	7 (47)
WHO performance status			
0	15 (41)	10 (48)	5 (33)
1–2	22 (59)	11 (52)	10 (69)
CIRS stage			
Primary	4 (11)	1 (5)	2 (13)
Intermediate	15 (41)	7 (33)	8 (53)
Secondary	18 (48)	13 (62)	5 (33)
Metastatic disease			
Metachronous	9 (24)	5 (24)	3 (20)
Synchronous	28 (76)	16 (76)	12 (80)
Primary tumor			
Colon	25 (68)	13 (62)	12 (80)
Rectum	12 (32)	8 (38)	3 (20)
Sites of metastases			
Liver	26 (70)	16 (76)	9 (60)
Lung	14 (38)	6 (29)	8 (53)
Lymph nodes	11 (30)	6 (29)	4 (27)
Local	7 (19)	2 (9)	4 (27)
Other	7 (19)	4 (19)	3 (20)
No. of involved sites			
1	14 (38)	9 (43)	5 (33)
≥2	23 (62)	12 (57)	10 (69)
Single metastatic sites			
Liver-limited	8 (22)	7 (33)	1 (7)
Other than liver	8 (22)	3 (14)	5 (33)
Lung	3 (8)	-	3 (20)
Lymph nodes	1 (3)	1 (5)	-
Local	4 (11)	2 (9)	2 (13)
Multiple metastatic sites	21 (57)	11 (52)	9 (60)
Liver metastases			
Single	3 (8)	3 (14)	-
Multiple	23 (62)	13 (62)	9 (60)
Previous adjuvant chemotherapy:	7 (19)	2 (9)	5 (33)
FA/5-FU bolus	1 (3)	1 (5)	-
XelOx or 5-FU/OXP	6 (16)	1 (5)	5 (33)
Previous radiotherapy:	4 (11)	3 (14)	1 (7)
RT+CT (5-FU continous infusion)	3 (8)	2 (9)	1 (7)
RT+CT (XELOX)	1 (3)	1 (5)	-

WHO, World Health Organization; CIRS, Cumulative Illness Rating Scale.

**Table:** B, Age and comorbidity stage in unfit patients

Age	Cumulative illness rating scale (CIRS)			Total no. (%)

	Primary	Intermediate	Secondary	
Non-elderly	2	5	2	9 (24)
Young-elderly	1	3	4	8 (22)
Old-elderly	1	7	12	20 (54)
Total no. (%)	4 (11)	15 (40)	18 (49)	37

**Table:** A, Activity, efficacy and effectiveness of first line regimens in unfit patients according to *KRAS* genotype

	All treated Intent-to-treat Analysis		*KRAS* wild-type Intent-to-treat Analysis		*KRAS* mutant Intent-to-treat Analysis	
		
	No.	%	No.	%	No.	%
Enrolled patients	37	100	21	100	15	100
Evaluable patients	27	70	14	67	12	80
Objective response	10	37 (CI ± 19)	7	50 (CI ± 27)	3	25 (CI ± 26)
Partial response	9	33	6	43	3	25
Complete response	1	4	1	7	-	-
Stable disease	9	33	4	29	5	42
Progressive disease	8	30	3	21	4	33
Median PFS, months	7		8		6	
Range	1−13+		1+−13+		1–11	
Progression events	28	76	15	71	12	80
Median OS, months	13		13		9	
Range	1+−23+		1+−23+		3–18	
Deaths	22	59	11	52	10	67
Liver metastasectomies	3		3		-	
No/overall pts	3/37	8	3/21	14	-	-
No/patients with liver metastases	3/26	11.5	3/16	19	-	-
No/patients with L-L metastases	3/8	37.5	3/7	43	-	-
Pathologic complete responses	1	33	1	33	-	-

PFS, progression-free survival; OS, overall survival, L-L, liver-limited.

**Table:** B, Activity, efficacy and effectiveness according to first line treatments

	Intent-to-treat analysis			

	Triplet regimen		Doublet regimen	
	
	No.	%	No.	%
Enrolled patients	18	100	15	100
Evaluable patients	16	89	9	60
Objective response	6	37.5 (CI ± 24)	4	44 (CI ± 34)
Partial response	6	37.5	3	33
Complete response	-	-	1	11
Stable disease	5	31	3	33
Progressive disease	5	31	2	22
Median PFS, months	8		8	
Range	3–12		1−13+	
Progression events	14	78	9	60
Median OS, months	12		15	
Range	3−23+		1+−23+	
Deaths	12	67	7	47
Liver metastasectomies	1	6	1	11
Pathologic complete responses	1	100	-	-

PFS, progression-free survival; OS, overall survival.
